# Supplementary material for: Patterns of Intron Gain and Loss in Fungi
Source: PLoS Biol. 2004 Nov 30;2(12):e422. doi: 10.1371/journal.pbio.0020422 (PMC532390; doi:10.1371/journal.pbio.0020422)
Supplement: Table S1 — Also available at http://genes.mit.edu/NielsenEtAl/. (4.3 MB ZIP). [file pbio.0020422.st001.zip › NielsenEtAl/html/526.html]

AN2085.1.NCU08605.1.MG04529.1.FG00367.1


```
 CLUSTAL W (1.82) Multiple Sequence Alignments - Introns Inserted


Sequence 1: NCU08605.1	275 aa
Sequence 2: MG04529.1	271 aa
Sequence 3: FG00367.1	270 aa
Sequence 4: AN2085.1	272 aa
Alignment Length: 276 aa
Number Identitical Residues: 188 aa
Alignment Score (without introns) 8192


MG04529.1 	MPGFDFSNVNRNNALHARGVPLPKATSTGTTIVGCIFEGGVV0IAADTRATSGPIVADK~
NCU08605.1	MPGFDFSNYNRNAALHARGVPLPKATSTGTTIVGCIYDGGVV0IAADTRATSGPIVADK~
FG00367.1 	MPGFDFSNYNRNAALHARGVPLPKATSTGTTIVGCIFDGGVV0IAADTRATSGPIVADK~
AN2085.1  	MAGFDFSNYNRNAALHAKGAPLPKATSTGTTIVGCIFDNGVV0IAADTRATSGPIVADK0
          	*.****** *** ****:*.****************::.*** **************** 

MG04529.1 	NCEKLHYISPQIWCAGAGTAADTEFTTAIISSQLELHSLSTGRKPRVVTCMTMLKQHLFQ
NCU08605.1	NCEKLHFIAPNIWCAGAGTAADTEFTTALISSQLELHSLSTGRKPRVVTCMTMLKQHLFR
FG00367.1 	NCEKLHYISPQIWCAGAGTAADTEFTTALISSQLELHSLSTGRKPRVVTCMTLLKQHLFR
AN2085.1  	NCEKLHYIAPKIWCAGAGTAADTEFTTALISSNIELHSLSTGRPPRVITCMTMLKQHLFR
          	******:*:*:*****************:***::********* ***:****:******:

MG04529.1 	YQGHIGAYLVVAGVDPTGTHLFTVHAHGSTDKLPYVTMGSGSLAAMSVFETQWQSKLDEA
NCU08605.1	YQGHIGAYLVVAGVDPTGTHLFTVHAHGSTDKLPYVTMGSGSLAAMSVFETQWKPQLNKE
FG00367.1 	YQGHIGAYLVVAGVDPTGTHLFTVHAHGSTDKLPYVTMGSGSLAAMSVFETQWKPDLSQE
AN2085.1  	YQGYIGAYLVVAGVDPTGIGLYTVHAHGSTDKLPYVTMGSGSLAAMSVFESTWKPNLNKE
          	***:**************  *:****************************: *:..*.: 

MG04529.1 	QAIKLCSDAIQAGIWNDLGSGSNVDVAVITADK-TRLLRNYIKPNERTKKLQSYKFARGT
NCU08605.1	EAMKLCSQAIEAGIWNDLGSGSNVDLAVITPEK-TTLHRGFVKPNERTQKLKSYKFKRGT
FG00367.1 	EAIKLASDAILAGVWNDLGSGSNVDVAIITSDK-TTLKRNYITPNQKEPKLQSYRFPKGT
AN2085.1  	EAIALASEAIKAGIFNDLGSGSNVDVCVIEKDKPTQLLRNYMRPNERGQKERDYRFPKGT
          	:*: *.*:** **::**********:.:*  :*.* * *.:: **::  * :.*:* :**

MG04529.1 	TAVLNDKIVKKEELSNFISVS----EVPAEGESMDVDS
NCU08605.1	TAVLNEKVIKKEEMGNYISVVELAEETPASGDKMEVDT
FG00367.1 	TAVLNEKIIKKEEIKRYITVE----DVPVE-EKMDVDT
AN2085.1  	TAYLNEKVFTKEDLRKYVTVE----EISGDPNLMEVDP
          	** **:*:..**:: .:::*     : . . : *:**.
```
